# miR-16-5p Regulates Proliferation and Apoptosis in High Glucose–Treated Human Retinal Microvascular Endothelial Cells by Targeting VEGFA and TGFBR1

**DOI:** 10.1155/joph/3082206

**Published:** 2025-03-24

**Authors:** JianFeng Zhao, YanFei Zhang, Yuan Xia, Jie Zhou, Yu Geng, HaiRong Hua

**Affiliations:** ^1^Department of Ophthalmology, First Affiliated Hospital of Kunming Medical University, Kunming 650032, Yunnan, China; ^2^Department of Pathology, Kunming Medical University, Kunming 650500, Yunnan, China

**Keywords:** diabetic retinopathy, miR-16-5p, proliferation, retinal microvascular endothelial cells, TGFBR1, VEGFA

## Abstract

Diabetic retinopathy (DR) is a common complication of diabetes and the main cause of vision loss in the middle-aged and elderly people. miRNAs play vital roles in the development of DR. This study aimed to explore the effects of miR-16-5p on high glucose (HG)–stimulated human retinal microvascular endothelial cells (HRECs) by modulating vascular endothelial growth factor A (VEGFA) and transforming growth factor beta receptor 1 (TGFBR1). HRECs were treated with 5 mM, 10 mM, 20 mM, and 30 mM of HG to induce the DR cell model. Real-time quantitative polymerase chain reaction (RT-qPCR) was used to detect the expression of miR-16-5p and mRNAs of VEGFA and TGFBR1. Western blot was used to examine VEGFA and TGFBR1 protein levels. The 3-(4, 5-dimethyl-2-thiazolyl)-2, 5-diphenyl-2-H-tetrazolium bromide assay was conducted to test cell proliferation. Flow cytometry with Annexin V-FITC/PI double staining was carried out to assess cell apoptosis ratio. Dual-luciferase assay was used to identify the target relationship between miR-16-5p and VEGFA and TGFBR1. Results found that the expression of miR-16-5p in HG-treated HRECs was reduced, and VEGFA and TGFBR1 expressions were upregulated. Knockdown of miR-16-5p increased VEGFA and TGFBR1 mRNA and protein levels, promoted cell proliferation, and inhibited apoptosis in HG-treated HRECs. VEGFA and TGFBR1 inhibition reversed the effect of knocking down miR-16-5p on HRECs. Dual-luciferase reporter assay revealed that VEGFA and TGFBR1 were the target of miR-16-5p. Overall, knockdown of miR-16-5p enhances proliferation and inhibits apoptosis of HRECs by upregulating VEGFA and TGFBR1 expression.

**Summary**

1. Overexpression of miR-16-5p inhibits HG-treated HREC proliferation
2. miR-16-5p targets VEGFA and TGFBR1
3. VEGFA and TGFBR1 inhibition reverses the effects of miR-16-5p inhibition on HRECs

## 1. Introduction

Diabetic retinopathy (DR) is a complication of diabetes mellitus. People with diabetes suffer from systemic microcirculation disturbance due to long-term elevated blood glucose and metabolic disorders in the body, and retinal vessels in the fundus are easily damaged, resulting in DR [1]. According to reports, about one-third of diabetic patients may develop into DR, which leads to the severe vision loss for patients [2]. Pathologic manifestations of DR include blood-retinal barrier rupture, pericyte loss, endothelial dysfunction, and vascular endothelial growth factor (VEGF)–associated retinal microangiopathy such as neovascularization, retinal edema, and diabetic macular edema [3, 4]. Retinal microvascular endothelial cells are the main constituent cells of retinal microvessels and play an important role in maintaining vascular tone, regulating blood pressure, antithrombosis, and angiogenesis [5]. The dysfunction of retinal vascular endothelial cells is closely related to DR [6, 7]. Therefore, exploring the molecular mechanisms regulating the biological function of retinal microvascular endothelial cells is crucial to elucidating the pathogenesis of DR.

miRNAs are noncoding RNAs that contain 20 ∼ 23 nucleotides. Most of the miRNAs found in plants, animals, and fungi are expressed in specific tissues and developmental stages [8, 9]. The tissue specificity and temporality of miRNA determine the functional specificity of tissue and cell, suggesting that miRNAs play multiple roles in the regulation of cell growth and development processes. miRNA regulates gene expression at the post-transcriptional level by degrading or inhibiting messenger RNA (mRNA) [10, 11]. Studies have found that miRNAs mediate the occurrence and development of a variety of diseases by regulating cellular physiological processes such as cell differentiation, proliferation, metastasis, and apoptosis [12, 13]. miRNAs are also involved in the regulation of DR. Li et al. [14] reported that inhibition of miR-200c-3p suppressed pyroptosis of high glucose (HG)–treated human retinal microvascular endothelial cells (HRECs). miR-16-5p is involved in cancers [15], osteoporosis [16], and many other diseases. Duan et al. [17] revealed that miR-16-5p improved HG-stimulated podocyte injury. However, the effect of miR-16-5p in DR remains unclear.

It has been found that vascular endothelial growth factor A (VEGFA) and transforming growth factor beta receptor 1 (TGFBR1) are upregulated in diabetes [18, 19]. In this study, we found that VEGFA and TGFBR1 are the targets of miR-16-5p through the prediction of Starbase database, and knockdown of miR-16-5p increased cell proliferation and inhibited apoptosis by upregulating VEGFA and TGFBR1 levels.

## 2. Materials and Methods

### 2.1. Cell Culture

Primary HRECs were provided by Hefei All Things Biological Technology Co., LTD (Anhui, China) (Cat: Delf-10746). HRECs were grown in HREC complete medium (Procell, Wuhan, China) and placed in a cell incubator at 37°C with 5% CO_2_. The third and fourth generation cells were used in the experiment.

### 2.2. Immunofluorescence (IF) Staining

HRECs were seeded into a 6-well plate. After adhering growth, the cells were inoculated on a cover glass and fixed with 4% paraformaldehyde. The cells were incubated with 0.1% Triton X-100 for 10 min and blocked with 10% goat serum for 2 h. The cells were incubated with anti-vWF and CD31 antibodies (Abcam, Shanghai, China) at 4°C overnight and incubated with the second antibody (Abcam, Shanghai, China) at room temperature for 1 h. The cells were sealed with DAPI and observed under a fluorescence microscope.

### 2.3. Cell Treatment and Transfection

HRECs were treated with 5 mM (normal glucose, NG group), 10 mM, 20 mM, and 30 mM glucose for 24 h. miR-16-5p mimic, miR-16-5p inhibitor, si-VEGFA, and si-TGFBR1 were synthesized by RIBOBIO (Guangzhou, China). HRECs were inoculated into 6-well plates (1 × 10^6^/well). According to the manufacturer's instructions of Lipofectamine 2000 (Merck Millipore, MA, USA), miR-16-5p mimic (5 nM), miR-16-5p inhibitor (100 nM), si-VEGFA (50 nM), si-TGFBR1 (50 nM), and the corresponding negative control were transfected into HRECs after treatment with 30 mM HG. After 48 h, real-time quantitative polymerase chain reaction (RT-qPCR) or western blot was performed to detect the transfection efficiency.

### 2.4. RT-qPCR

Total RNA was extracted using TRIzol reagent (Thermo Fisher Scientific, MA, USA). Reverse transcription was performed using a reverse transcription kit (Biomarker Technologies, Beijing, China). A SYBR-Green PCR MasterMix kit (Thermo Fisher Scientific, MA, USA) was used to conduct RT-qPCR. The relative expressions of VEGFA, TGFBR1 mRNA, and miR-16-5p were quantified by the 2^−ΔΔCt^ method. U6 served as the internal reference of miR-16-5p, and GAPDH served as the internal reference of VEGFA and TGFBR1. The primer sequences are shown in Table 1.

### 2.5. Western Blot

RIPA was applied to extract the total protein, and 20 μg of protein samples was used for western blot detection. The protein was separated by sodium dodecyl sulfate - polyacrylamide gel electrophoresis gel electrophoresis and transferred to polyvinylidene fluoride membranes, then the membranes were blocked in 5% skim milk for 2 h. The membranes were incubated with primary antibody (Abcam, Shanghai, China) and the secondary (Abcam, Shanghai, China) antibody. The membranes were visualized using enhanced chemiluminescence reagent, and the relative expression levels of VEGFA and TGFBR1 protein were analyzed using ImageJ software.

### 2.6. 3-(4, 5-Dimethyl-2-thiazolyl)-2, 5-Diphenyl-2-H-tetrazolium Bromide (MTT) Assay

MTT assay was used to detect the proliferation ability of cells. Cells were cultured in a 96-well plate (5 × 10^3^ cells/well) for 48 h. MTT solution (Yeasen Biotechnology (Shanghai) Co., Ltd.) was added to each well and incubated for 4 h. 150 μL of dimethyl sulfoxide (Jiangsu Pules Biotechnology Co., Ltd.) was added to each well to dissolve the formazan. The optical density (OD) value at 490 nm was measured using a microplate reader.

### 2.7. Flow Cytometry

Apoptosis ratio was assessed by a Annexin V FITC/PI double staining kit (Beyotime, Shanghai, China). Briefly, cells were digested with 0.25% trypsin, rinsed with PBS, and resuspended using Annexin V-FITC binding buffer. 5 μL of Annexin V-FITC and 2 μL of PI were added to cells and incubated at room temperature for 15 min and 5 min. The apoptotic rate was detected by flow cytometry.

### 2.8. Dual-Luciferase Reporter Gene Assay

The wild-type (WT) and mutant (MUT) sequences of VEGFA and TGFBR1 were synthesized and inserted into the pmiR-GLO vector to obtain a WT/MUT plasmid of VEGFA and TGFBR1. HRECs were collected and cultured in a 24-well plate (1 × 10^4^/well). The plasmids, NC mimic, and miR-16-5p mimic were transfected into HRECs. After culturing for 48 h, the total RNA was extracted, and the dual-luciferase activity was detected by the dual-luciferase reporter gene system (MedChem Express, NJ, USA) according to the manufacturer's instructions.

### 2.9. Statistical Analysis

The data were presented as “mean ± standard deviation.” GraphPad Prism 8.0 software was used to analyze data and graph. The differences between two groups were analyzed using Student's *t*-test. The differences among multiple groups were analyzed using one-way ANOVA. *p* < 0.05 was considered statistically significant.

## 3. Results

### 3.1. The Expression of miR-16-5p, VEGFA, and TGFBR1 in HG-Treated HRECs

**Figure 1:**
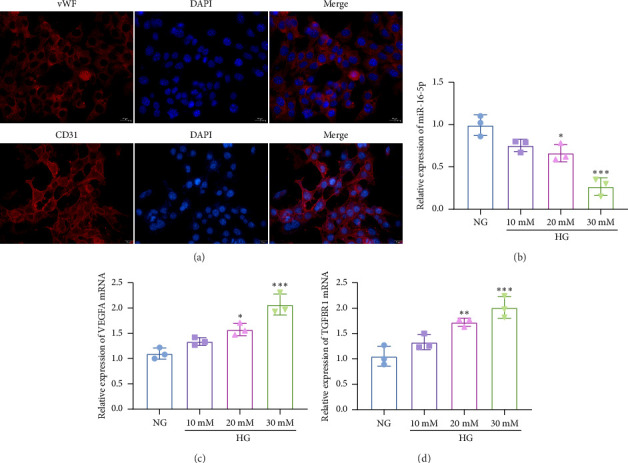
The expression of miR-16-5p, VEGFA, and TGFBR1 in HG-treated HRECs. (a) The fluorescence images of HREC markers vWF and CD31 (scale bar: 20 μm). RT-qPCR was performed to assess the expression of miR-16-5p (b), VEGFA (c), and TGFBR1 (d) mRNA in HG-stimulated HRECs. Compared with NG group, ⁣^∗^*p* < 0.05, ⁣^∗∗^*p* < 0.01, ⁣^∗∗∗^*p* < 0.001. *n* = 3 independent experiments.

The HREC markers vWF and CD31 were significantly expressed (Figure 1(a)) in HRECs. DR cell models were established by treating HRECs with 10 mM, 20 mM, or 30 mM glucose, respectively. RT-qPCR was used to examine miR-16-5p and the mRNAs expression of VEGFA and TGFBR1. The results found that miR-16-5p was decreased with the increase of glucose concentration (Figure 1(b)), and the expression was lowest at 30 mM. The expression of VEGFA (Figure 1(c)) and TGFBR1 (Figure 1(d)) mRNAs was increased with the increase of glucose concentration.

### 3.2. miR-16-5p Regulates Cell Proliferation and Apoptosis in HG-Induced HRECs

**Figure 2:**
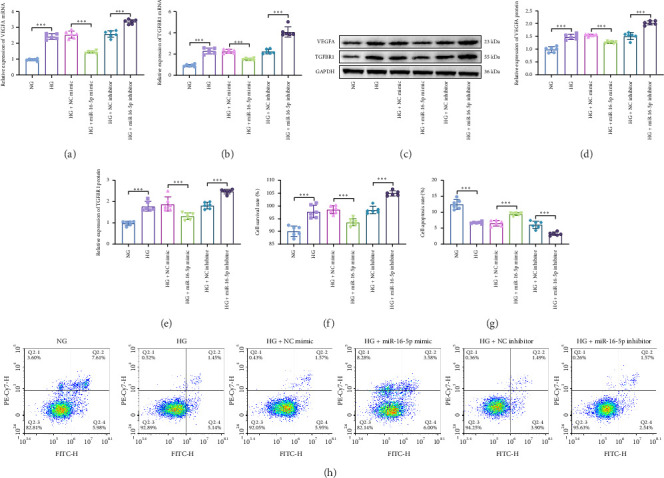
miR-16-5p regulates cell proliferation and apoptosis in HG-induced HRECs. The expression of VEGFA (a) and TGFBR1 (b) mRNAs was detected using RT-qPCR. (c, d, e) Western blot was used to detect VEGFA and TGFBR1 proteins. (f) MTT was used to determine cell proliferation. (g, h) The apoptosis rate of HRECs was tested by flow cytometry. ⁣^∗^*p* < 0.05, ⁣^∗∗^*p* < 0.01, ⁣^∗∗∗^*p* < 0.001. *n* = 6 independent experiments.

The HRECs stimulated by 30 mM HG were transfected with miR-16-5p mimic, miR-16-5p inhibitor, and their corresponding negative controls. VEGFA and TGFBR1 mRNA (Figures 2(a) and 2(b)) and protein (Figures 2(c), 2(d), and 2(e)) levels were increased in the HG group in comparison with the NG group. Compared with the HG + NC mimic group, VEGFA and TGFBR1 mRNA and protein levels in miR-16-5p mimic group decreased, and miR-16-5p inhibitor promoted VEGFA and TGFBR1 mRNA and protein levels than those in the HG + NC inhibitor group. MTT and flow cytometry assays revealed that treatment with HG enhanced cell proliferation (Figure 2(f)) and decreased apoptosis (Figures 2(g) and 2(h)) in HRECs, overexpression of miR-16-5p decreased cell proliferation and increased apoptosis, and knockdown of miR-16-5p promoted cell proliferation and inhibited HREC apoptosis. These results reveal that miR-16-5p negatively regulates the expression of VEGFA and TGFBR1, and overexpression of miR-16-5p may be a potential therapeutic target for DR.

### 3.3. miR-16-5p Targets VEGFA and TGFBR1

**Figure 3:**
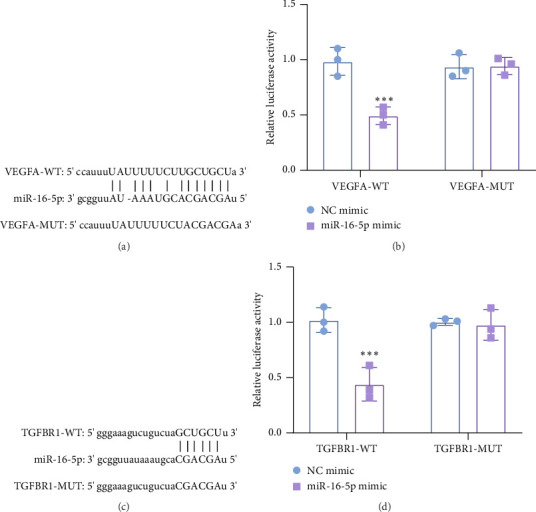
miR-16-5p targets VEGFA and TGFBR1. (a) Starbase database predicted the binding sequences between miR-16-5p and VEGFA. (b) Dual-luciferase reporter assay was performed to verify the target relationship between miR-16-5p and VEGFA. (c) Starbase database predicted the binding sequences between miR-16-5p and TGFBR1. (d) Dual-luciferase reporter assay was performed to verify the target relationship between miR-16-5p and TGFBR1. Compared with NC mimic group, ⁣^∗∗∗^*p* < 0.001. *n* = 3 independent experiments.

VEGFA was a potential target of miR-16-5p predicted by Starbase database (Figure 3(a)). Dual-luciferase reporter assay found that compared with NC mimic group, miR-16-5p mimic inhibited the luciferase activity of WT VEGFA (Figure 3(b)), and there was no significant effect on MUT VEGFA. Meanwhile, the Starbase database predicted that miR-16-5p has binding sequences with the 3′untranslated region of TGFBR1 (Figure 3(c)). Dual-luciferase indicated that miR-16-5p mimic suppressed the luciferase activity of WT of TGFBR1 (Figure 3(d)). These results demonstrate that miR-16-5p targets VEGFA and TGFBR1.

### 3.4. Knockdown of miR-16-5p Regulates Proliferation and Apoptosis of HG-Treated HRECs Through VEGFA and TGFBR1

**Figure 4:**
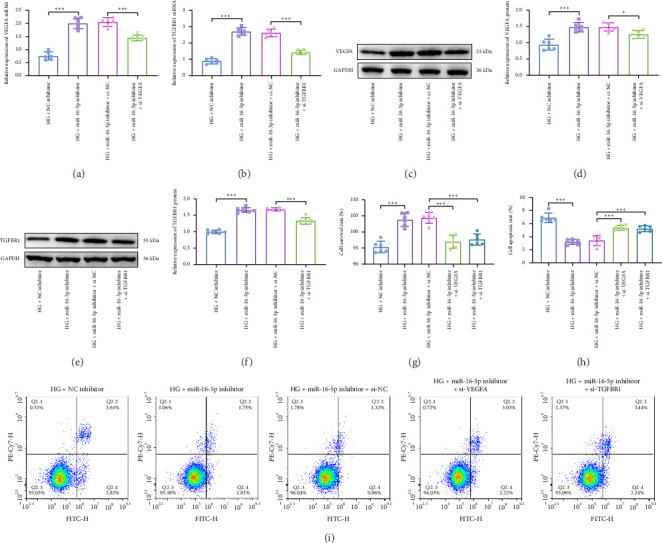
Knockdown of miR-16-5p regulates proliferation and apoptosis of HG-treated HRECs through VEGFA and TGFBR1. RT-qPCR was carried out to detect the expression of VEGFA (a) and TGFBR1 mRNA (b). Western blot was used to measure VEGFA (c, d) and TGFBR1 (e, f) protein levels. (g) MTT was performed to detect cell proliferation. (h, i) Flow cytometry was used to test the apoptosis rate of HRECs. ⁣^∗^*p* < 0.05, ⁣^∗∗^*p* < 0.01, ⁣^∗∗∗^*p* < 0.001. *n* = 6 independent experiments.

Next, the roles of miR-16-5p/VEGFA/TGFBR1 axis in HG-induced HRECs were investigated. NC inhibitor, miR-16-5p inhibitor, si-NC, si-VEGFA, and si-TGFBR1 were transfected into HRECs. Inhibition of miR-16-5p increased VEGFA and TGFBR1 mRNA (Figures 4(a) and 4(b)) and protein levels (Figures 4(c), 4(d), 4(e), and 4(f)). Transfection with si-VEGFA suppressed VEGFA mRNA (Figure 4(a)) and protein expression (Figures 4(c) and 4(d)), and transfection with si-TGFBR1 decreased TGFBR1 mRNA (Figure 4(b)) and protein expression (Figures 4(e) and 4(f)) in HRECs. MTT and flow cytometry assays results indicated that compared with knockdown of miR-16-5p group, knockdown of VEGFA or TGFBR1 inhibited cell proliferation (Figure 4(g)) and promoted apoptosis (Figures 4(h) and 4(i)). Taken together, knockdown of miR-16-5p increases proliferation and suppresses apoptosis through promoting VEGFA and TGFBR1 expression.

## 4. Discussion

DR is the most common complication of diabetes and the leading cause of blindness in adults. Early detection and timely treatment are essential to delay its progression and preserve existing eyesight in most patients. The current clinical treatments of DR include laser therapy and antiangiogenic factor drug therapy (such as aflibercept, bevacizumab, and ranibizumab) [20, 21]. However, the treatment is not ideal, and there are side effects. Therefore, it is momentous to clarify the molecular mechanism of DR and develop new therapeutic strategies. Here, we found that miR-16-5p expression was decreased in HG-stimulated HRECs, and downregulation of miR-16-5p promoted proliferation of HG-stimulated HRECs and dampened cell apoptosis by targeting VEGFA and TGFBR1.

miRNAs are key regulatory genes in the development process of DR. Kovacs et al. [22] analyzed miRNA expression profiles in normal and diabetic rat retinas and RECs, and results found that 86 and 120 miRNAs are differentially expressed in the diabetic and normal groups. Knockdown of miR-200c-3p mitigated HG-treated HRECs pyroptosis and inhibited the progression [14]. Knockdown of miR-139-5p eliminated the cell migration and tube formation of HRMECs, decreased the acellular capillaries, and suppressed the formation of aberrant blood vessels in the retinal tissues [23]. Knockdown of miR-423-5p suppressed HG-treated cell proliferation, migration, and angiogenesis [24]. In the present study, we revealed that treatment with HG promoted proliferation of HRECs and inhibited apoptosis. Overexpression of miR-16-5p dampened cell proliferation and induced apoptosis rate in HREC treatment with 30 mM HG. Knockdown of miR-16-5p promoted VEGFA and TGFBR1 expressions, promoted HG-treated HRECs proliferation, and inhibited apoptosis. However, the role of HG on cell proliferation has an inconsistent report. Castilho et al. [25] found that long-term exposure of human umbilical vascular endothelial cells to HG (30 μM) decreases cell viability. HG at 40 mM was significantly cytotoxic and reduced cell viability of HRECs [26]. Overexpression of miR-16 increased IGFBP3 levels by decreasing TNF-α and SOCS3 signaling, inhibited insulin resistance, and protected retinal endothelial cells from HG-treated apoptosis [27]. miR-16-5p is also involved in the regulation of other diseases. Overexpressed miR-16-5p suppressed cell proliferation, migration, and induced apoptosis of breast cancer cells [15]. Overexpression of miR-16-5p inhibited bleomycin-induced skin fibrosis and reduced dermal thickening [28].

It was reported that VEGFA regulates tumors [29], idiopathic membranous nephropathy [30], and osteoporosis [31] through modulating angiogenesis. Inhibition of VEGFA transcription significantly inhibited angiogenesis in triple negative breast cancer mice and improved the survival rate of mice [32]. Ke et al. [30] reported that VEGFA induces the occurrence of PLA2R-associated idiopathic membranous nephropathy by angiogenesis. Upregulation of VEGFA induced angiogenesis and may serve as a novel target for treating osteoporosis [31]. In this study, we found that VEGFA expression in HG-treated HRECs was increased, and knockdown of VEGFA reversed the effect of knockdown miR-16-5p on HRECs, inhibited cell proliferation, and promoted apoptosis.

TGFBR1 is located at 9q22.33 and a key receptor of TGF-β/Smad signaling pathway [33]. TGFBR1 gene mutation mediates the development of cancers, myocardial fibrosis, and pulmonary fibrosis [34–36]. For example, upregulation of TGFBR1 transcript enhanced the proliferation, invasion, and migration ability of non-small-cell lung cancer cells [34]. Inhibition of TGFBR1 signaling pathway reduced myocardial fibrosis and cardiac dysfunction in mice, and improved cardiac function in mice with myocardial infarction [35]. Decreased TGFBR1 exerted an antifibrotic effect in silica-induced pulmonary fibrosis by suppressing TGF-β1 signaling pathway [36]. Inhibition of TGFBR1 signaling pathway ameliorated kidney injury in diabetic nephropathy [37]. In diabetic cataract, overexpression of TGFBR1 promoted the migration and epithelial–mesenchymal transition in HG-treated lens epithelial cells. Here, we revealed that TGFBR1 was a target of miR-16-5p, and knockdown of TGFBR1 repressed the proliferation and promoted apoptosis in HG-treated HRECs.

In conclusion, miR-16-5p was downregulated in HG-treated HRECs, and knockdown of miR-16-5p promoted cell proliferation and reduced apoptosis in HG-treated HRECs by upregulating VEGFA and TGFBR1 expression. miR-16-5p/VEGFA/TGFBR1 axis may serve as a promising target in the diagnosis and treatment of DR.

## Figures and Tables

**Table 1:** Primer sequences.

Genes	NCBI	Primer sequences (5′-3′, F: forward, R: reverse)
miR-16-5p	NC_000013.11	F: CGCGTAGCAGCACGTAAATA
		R: AGTGCAGGGTCCGAGGTATT

U6	NR_002083.1	F: ATTGGAACGATACAGAGAAGATT
		R: GGAACGCTTCACGAATTTG

VEGFA	NM_001317010.2	F: GCTCGGTGCTGGAATTTGAT
		R: AAAAGTTTCAGTGCGACGCC

TGFBR1	NM_001407423.1	F: TGAGCCTGTTGGAGGTTCAG
		R: ACAGCAACTTCTTCTCCCCG

GAPDH	NM_001357943.2	F: GTCTCCTCTGACTTCAACAGCG
		R: ACCACCCTGTTGCTGTAGCCAA

## Data Availability

The data underlying this article are available from the corresponding author upon request.
